# Author Correction: *Candida albicans* gains azole resistance by altering sphingolipid composition

**DOI:** 10.1038/s41467-019-08339-2

**Published:** 2019-01-15

**Authors:** Jiaxin Gao, Haitao Wang, Zeyao Li, Ada Hang-Heng Wong, Yi-Zheng Wang, Yahui Guo, Xin Lin, Guisheng Zeng, Yue Wang, Jianbin Wang

**Affiliations:** 1 School of Life Sciences, Tsinghua University, Beijing 100084, China; 2 Centre for Life Sciences, Tsinghua University, Beijing 100084, China; 3 Faculty of Health Sciences, University of Macau, Macau, China; 4 Institute of Molecular and Cell Biology, Agency for Science, Technology and Research, Singapore, 138673 Singapore; 5 Peking-Tsinghua-NIBS Joint Graduate Program, Tsinghua University, Beijing 100084, China; 6 Institute for Immunology, School of Medicine, Tsinghua University, Beijing 100084, China; 7 Department of Biochemistry, Yong Loo Lin School of Medicine, National University of Singapore, Singapore, 117596 Singapore

Correction to: *Nature Communications*; 10.1038/s41467-018-06944-1; published online 29 October 2018

In the original version of this Article, Haoping Liu, who conceptualized, designed and supervised the project and acquired funding, was inadvertently omitted from the author list. Furthermore, the affiliation of Jiaxin Gao and Haoping Liu with ‘Department of Biological Chemistry, University of California, Irvine, CA 92697, USA’ was omitted. Finally, funding from NIH grant GM117111, and contributions from Dr. Li-lin Du of NIBS for providing pPB[ura4] and pDUAL-PBase and Allan Bradley of Sanger for hyPBase, were not acknowledged. These errors have now been corrected in both the PDF and HTML versions of the Article.

