# Supplementary material for: Candida albicans gains azole resistance by altering sphingolipid composition
Source: Nat Commun. 2018 Oct 29;9:4495. doi: 10.1038/s41467-018-06944-1 (PMC6206040; doi:10.1038/s41467-018-06944-1)
Supplement: Supplementary file 1 — Supplementary Information [file 41467_2018_6944_MOESM1_ESM.pdf]

Supplementary Information

Candida albicans gains azole resistance by altering sphingolipid composition

Gao et al.

Description of Supplementary Files

File Name: Supplementary Information
Description: Supplementary Figures, Supplementary Tables and Supplementary References

File Name: Supplementary Data 1
Description: Oligonucleotide primers used in this study.

File Name: Supplementary Data 2
Description: Genome-wide profiling of genes whose inactivation require for fluconazole susceptibility.

Supplementary Figure 1

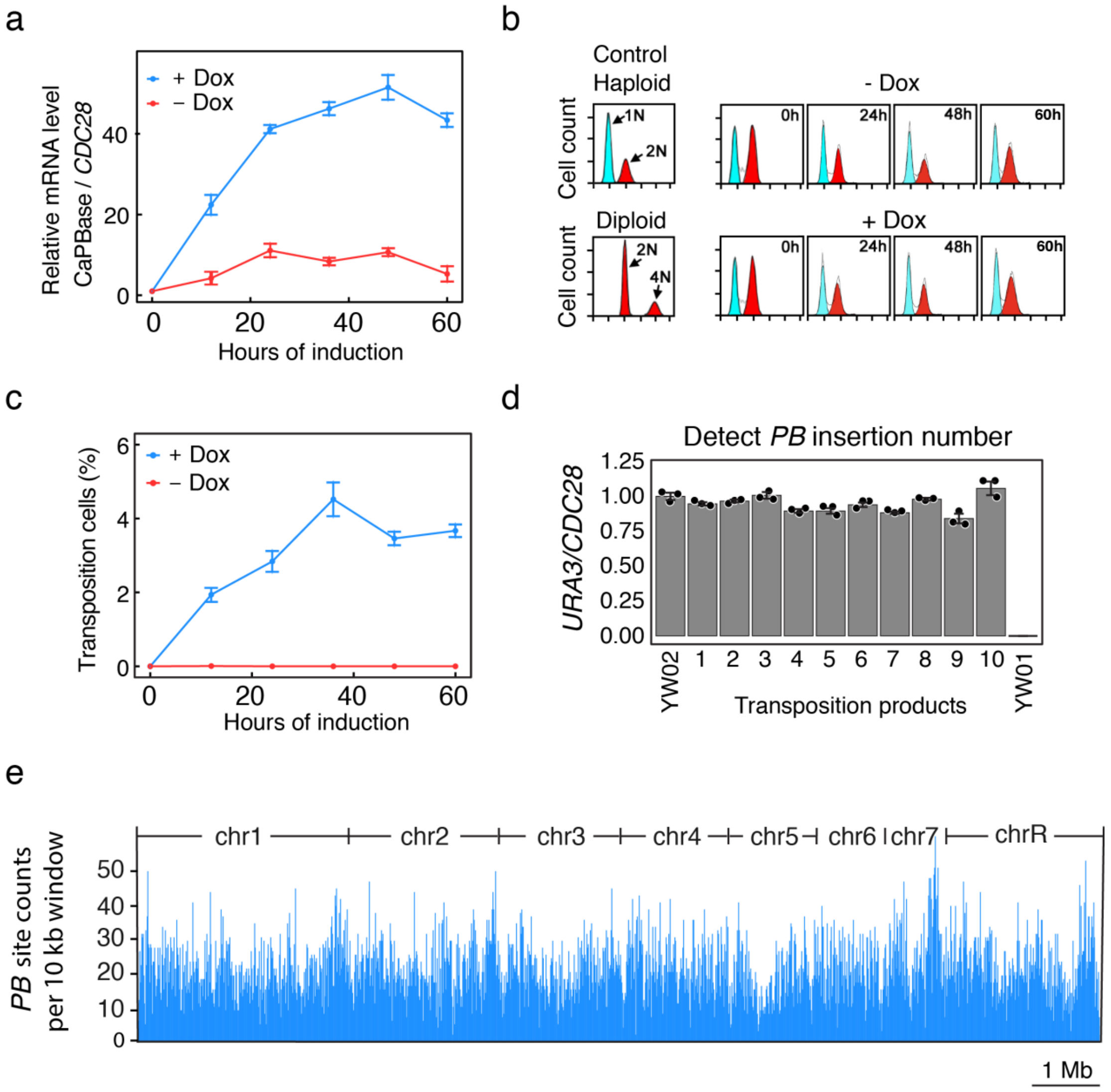

Supplementary Figure 1. Quantitative measurement of *PB* transposition in liquid medium.

(a) qPCR analysis of CaPBase expression level during transposition induction. *CDC28* mRNA level was used for normalization. Error bars represent standard deviation (s.d.) from the mean of triplicate samples.

(b) Ploidy analysis during transposition induction by flow cytometry. The DNA content of the induced cells at each time point was compared with that of haploid and diploid control strains.

(c) Estimation of transposition efficiency in YW02. Error bars represent s.d. from the mean of three independent experiments.

(d) Analysis of *PB* copy number in transposition derivatives by comparing the levels of *URA3* and *CDC28* DNA using qPCR. YW01 and YW02 were included as controls. Error bars represent s.d. from the mean of triplicate samples.

(e) NGS analysis of genome-wide *PB* insertion distribution. *PB*-specific insertion sites were plotted in 10-kb sliding windows.

Supplementary Figure 2

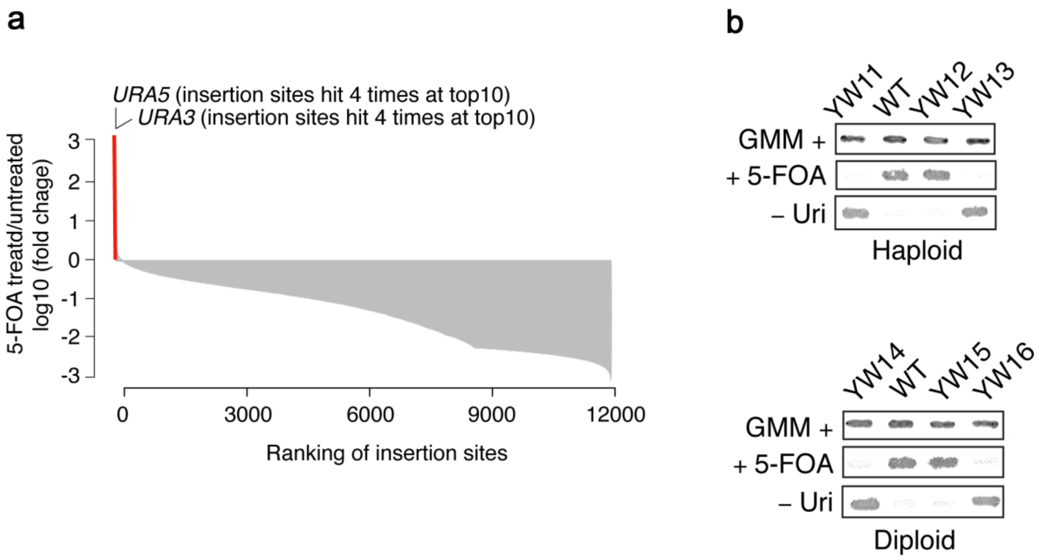

Supplementary Figure 2. Genetic screen of 5-FOA resistant mutants.

(a) Fold changes of insertion sites after growing the mutant library in media containing 5-FOA in comparison with the same library grown in normal media. The top ten insertions are highlighted in red.

(b) Confirmation of the auxotrophic phenotypes of haploid and diploid *URA5* deletion mutants. Strains grown on a GMM+Uri+Arg+His (GMM+) plate were replica-transferred onto a GMM+Uri+5-FOA (+5-FOA) or GMM+Arg+His (-Uri) plate and then incubated at 30°C overnight. GZY803 and BWP17 were included as controls.

Supplementary Figure 3

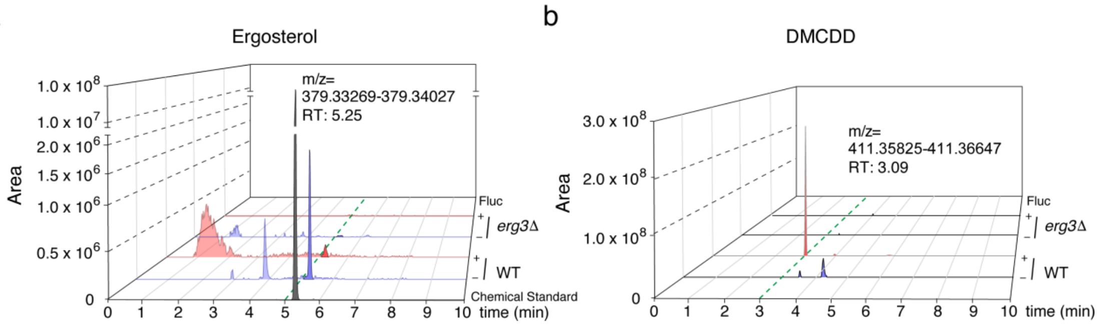

Supplementary Figure 3. Representative data of LC-MS analysis of sterols.

(a and b) LC-MS/MS base peak ion chromatograms of the ergosterol standard and sterols extracted from WT and *erg3*Δ cells with or without fluconazole treatment.

Supplementary Figure 4

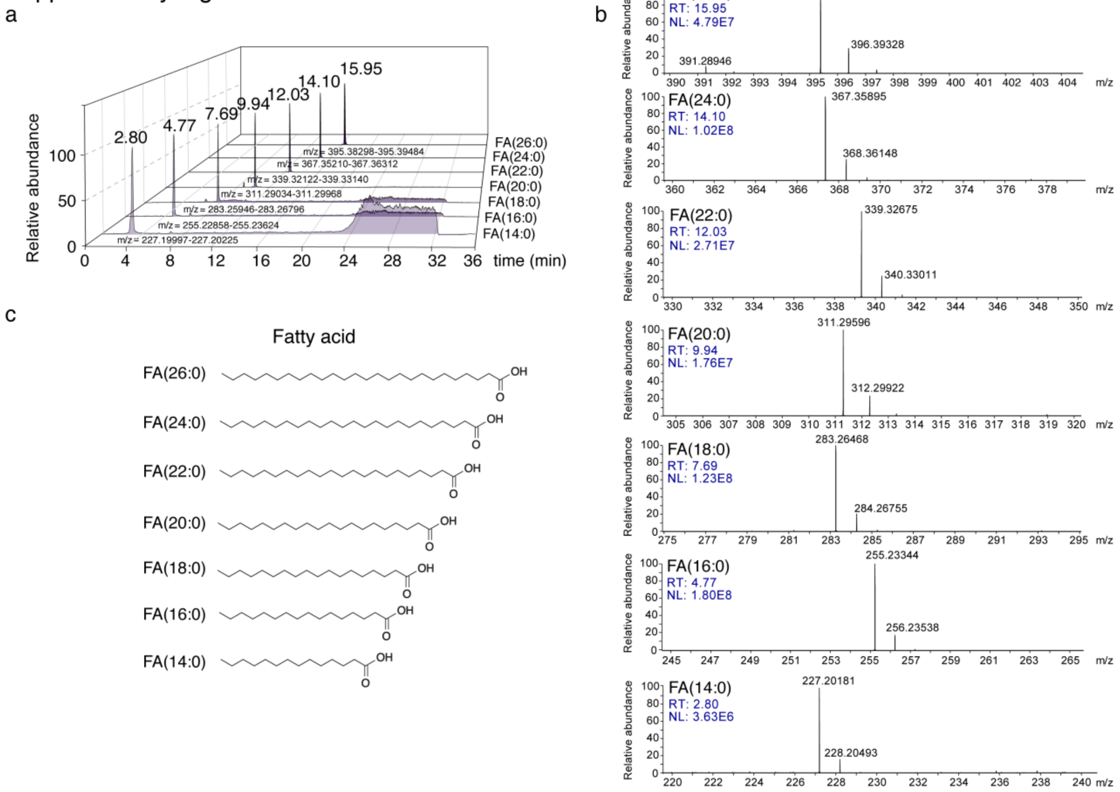

Supplementary Figure 4. Representative data of LC-MS analysis of free fatty acids.

(a and b) LC-MS spectra of free fatty acids extracted from WT, *fen1*Δ/Δ and *fen12*Δ/Δ cells.

(c) Structures of free fatty acid species detected by LC-MS.

Supplementary Figure 5

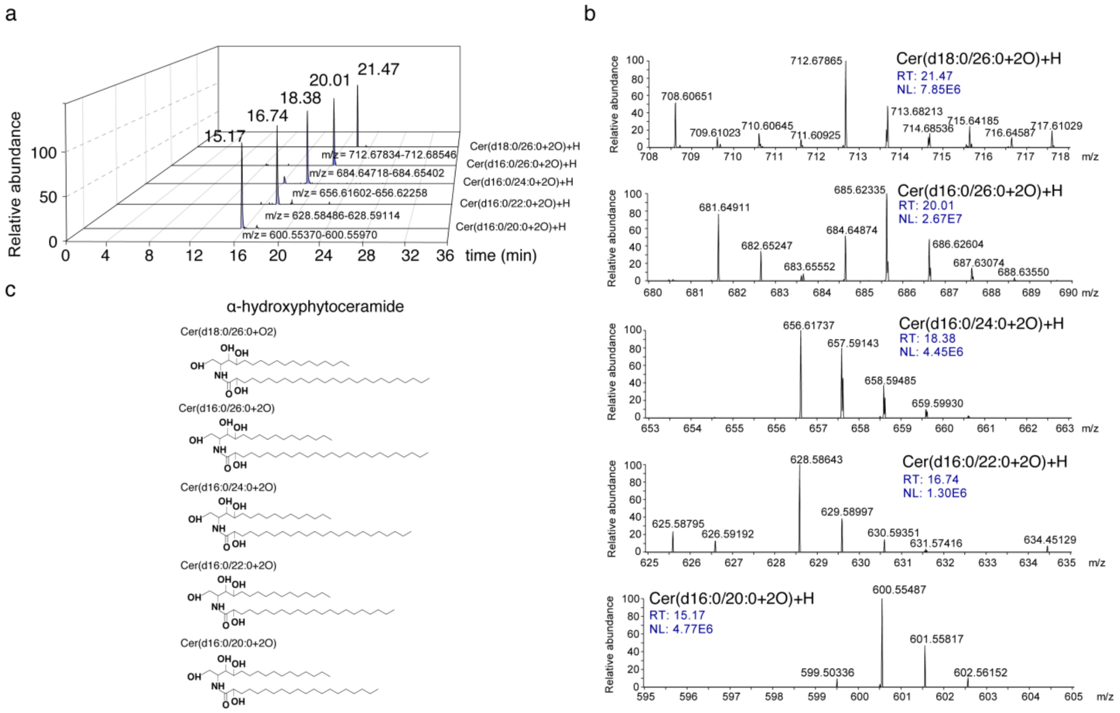

Supplementary Figure 5. Representative data of LC-MS analysis of α-hydroxyphytoceramide.

(a and b) LC-MS spectra of α-hydroxyphytoceramide extracted from WT, *fen1*Δ/Δ and *fen12*Δ/Δ cells.

(c) Structures of α-hydroxyphytoceramide species detected by LC-MS.

Supplementary Figure 6

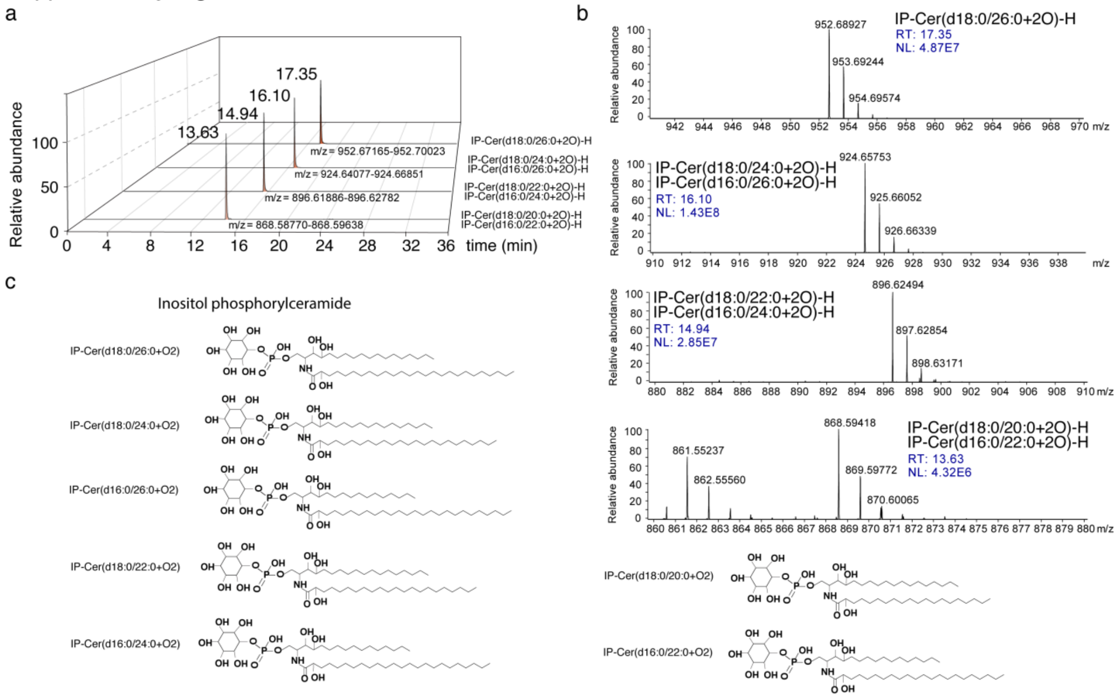

Supplementary Figure 6. Representative results of LC-MS analysis of inositol-phosphoceramide.

(a and b) LC-MS spectra of inositol-phosphoceramide extracted from WT, *fen1*Δ/Δ and *fen12*Δ/Δ cells.

(c) Structures of inositol-phosphoceramide detected by LC-MS.

Supplementary Figure 7

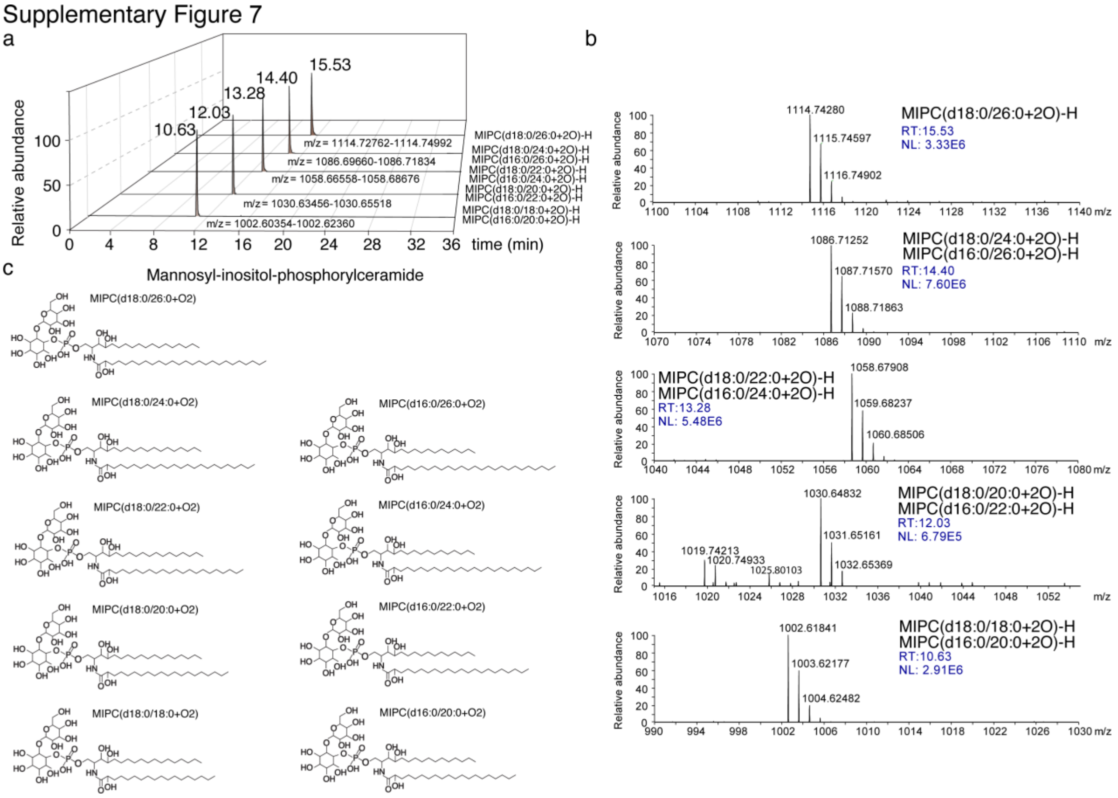

Supplementary Figure 7. Representative results of LC-MS analysis of mannosylinositol-phosphorylceramide.

(a and b) LC-MS spectra of mannosylinositol-phosphoceramide extracted from WT, *fen1*Δ/Δ and *fen12*Δ/Δ cells.

(c) Structures of mannosylinositol-phosphoceramide species detected by LC-MS.

Supplementary Table 1. Identification of *PB* insertion sites in the *C. albicans* genome.

Insertion locus	Coordinate in allele 'a'	Coordinate in allele 'b'	Insertion site
chr7 RVB2/C7_01810W (orf19.6539)	389861	389864	TGTACCAATGTTAA
chr5 APM1/C5_05400W (orf19.4036)	1167339	1167464	TGTTGTGATTTTAA
chr2 YWP1/C2_08590W (orf19.3618)	1746436	1746463	AGAATGGTGATTAA
chrR CR_05500C (orf19.5852)	1171307	1171235	ATTTTATTCATTAA
chr7 GRX1/C7_02070W (orf19.6510)	460971	460978	AAGTTATTTCTTAA
chr3 TRY4/C3_05050W (orf19.5975)	1087167	1087145	GTAATAGTTGTTAA
chr2 C2_07040W (orf19.2260)	1441078	1441095	AAAAAAAAAGTTAA
chr1 CDC5/C1_00950C (orf19.6010)	193757	193765	CTTTTTTTTTTTAA
chr1 C1_00830W (orf19.6023)	158466	158470	TATGATTTGATTAA
chr6 C6_03050C (orf19.5587)	641791	641749	CAAACTATTATTAA
chr3 C3_06950W (orf19.6805)	1593385	1593359	TTAACATTCCTTAA
chr2 C2_02020W (orf19.1516)	390042	390044	TTCCCATTGTTTAA
chr4 C4_01090C (orf19.4680)	223286	223317	AGAAAGCTCTTTAA
chr6 C6_00290W (orf19.1189)	41506	41515	CTCTAATCCCTTAA
chr1 SMT3/C1_11330C (orf19.670)	2487182	2487223	ACGTTTAACTTTAA
chr3 ULP1/C3_03550C (orf19.353)	755013	755004	GGATAATGGCTTAA
chr2 CAK1/C2_04270W (orf19.793)	896450	896482	ACCTTTTTTTTTAA
chr1 C1_00820W (orf19.6024)	158153	158157	AACATAATCATTAA
chr3 C3_07460W (orf19.6742)	1707077	1707049	TGTAAATCAATTAA
chr4 AGO1/C4_06340W (orf19.2903)	1409905	1409939	TTTTTTTTTATTAA

Supplementary Table 2. *C. albicans* strains used in this study.

Strains	Ploidy	Genotype	Source
SC5314	Diploid	WT	1
BWP17	Diploid	*ura3 /ura3 his1::hisG/his1::hisG arg4::hisG/arg4::hisG*	2
GZY803	Haploid	*MTLα his4 ura3Δ::HIS4*	3
YW01	Haploid	*MTLα his4 ura3Δ::HIS4* $P_{Tet\text{-}On}$*-CaPBase::SAT1*	This study
YW02	Haploid	*MTLα his4 ura3Δ::HIS4* $P_{Tet\text{-}On}$*-CaPBase::SAT1 ARG4::PB[URA3]* (TTAA, n.t. 709-712 within the ORF of *ARG4*, forward strand)	This study
YW05	Haploid	*MTLα his4 ura3Δ::HIS4* $P_{Tet\text{-}On}$*-CaPBase::SAT1 his1Δ::URA3 ARG4::PB[HIS1]* (TTAA, n.t. 709-712 within the ORF of *ARG4*, forward strand)	This study
YW06	Haploid	*MTLα his4 ura3Δ::HIS4* $P_{Tet\text{-}On}$*-CaPBase::SAT1 his1Δ::URA3* P_{URA3}*::PB[HIS1]* (TTAA, n.t. 104-107 upstream of start codon of *URA3*, forward strand)	This study
YW07	Haploid	*MTLα his4 ura3Δ::HIS4* $P_{Tet\text{-}On}$*-CaPBase::SAT1 his1Δ::URA3 URA3::PB[HIS1]* (TTAA, n.t. 76-79 within the ORF of *URA3*, forward strand)	This study
YW08	Haploid	*MTLα his4 ura3Δ::HIS4* $P_{Tet\text{-}On}$*-CaPBase::SAT1 his1Δ::URA3 URA3::PB[HIS1]* (TTAG, n.t. 238-241 within the ORF of *URA3*, forward strand)	This study
YW09	Haploid	*MTLα his4 ura3Δ::HIS4* $P_{Tet\text{-}On}$*-CaPBase::SAT1 his1Δ::URA3 URA3::PB[HIS1]* (TTAA, n.t. 76-79 within the ORF of *URA3*, reverse strand)	This study
YW10	Haploid	*MTLα his4 ura3Δ::HIS4* $P_{Tet\text{-}On}$*-CaPBase::SAT1 his1Δ::URA3 URA3::PB[HIS1]* (CTAA at n.t. 628-631 within the ORF of *URA3*, reverse strand)	This study
YW11	Haploid	*MTLα his4 ura3Δ::HIS4 his1Δ::URA3*	This study
YW12	Haploid	*MTLα his4 ura3Δ::HIS4 his1Δ::URA3 ura5Δ::HIS1*	This study
YW13	Haploid	*MTLα his4 ura3Δ::HIS4 his1Δ::URA3 ura5Δ::HIS1 URA5*	This study
YW14	Diploid	*BWP17 URA3*	This study
YW15	Diploid	*BWP17 URA3 ura5Δ::HIS1 ura5Δ::ARG4*	This study
YW16	Diploid	*BWP17 URA3 ura5Δ::HIS1 ura5Δ::ARG4 URA5*	This study
YW23	Haploid	*MTLα his4 ura3Δ::HIS4 ERG3Δ::URA3*	This study
YW24	Haploid	*MTLα his4 ura3Δ::HIS4 PMC1Δ::URA3*	This study
YW25	Haploid	*MTLα his4 ura3Δ::HIS4 C5_04050WΔ::URA3*	This study
YW26	Haploid	*MTLα his4 ura3Δ::HIS4 C6_03800CΔ::URA3*	This study
YW27	Haploid	*MTLα his4 ura3Δ::HIS4 MVB12Δ::URA3*	This study
YW28	Haploid	*MTLα his4 ura3Δ::HIS4 NOT5Δ::URA3*	This study
YW29	Haploid	*MTLα his4 ura3Δ::HIS4 FEN12Δ::URA3*	This study
YW30	Haploid	*MTLα his4 ura3Δ::HIS4 FMP27Δ::URA3*	This study
YW31	Haploid	*MTLα his4 ura3Δ::HIS4 C7_02530CΔ::URA3*	This study
YW32	Haploid	*MTLα his4 ura3Δ::HIS4 FGR32Δ::URA3*	This study
YW33	Diploid	*BWP17 FEN12Δ::URA3 FEN12Δ::HIS1*	This study
YW34	Diploid	*BWP17 FEN1Δ::UFP FEN1Δ::HIS1*	This study
YW35	Diploid	*BWP17 LAG1Δ::SAT1*	This study
YW36	Diploid	*BWP17 FEN12Δ::URA3 FEN12Δ::HIS1 LAG1Δ::ARG4*	This study
YW37	Diploid	*BWP17 FEN1Δ::UFP FEN1Δ::HIS1 LAG1Δ::ARG4*	This study
YW38	Diploid	*BWP17 UPC2Δ::ARG4 UPC2Δ::SAT1*	This study
YW39	Diploid	*BWP17 FEN12Δ::URA3 FEN12Δ::HIS1 UPC2Δ::ARG4 UPC2Δ::SAT1*	This study
YW40	Diploid	*BWP17 UPC2Δ::ARG4*	This study
YW41	Diploid	*BWP17 FEN12Δ::URA3 FEN12Δ::HIS1 UPC2Δ::SAT1*	This study
YW42	Diploid	*BWP17 FEN1Δ::UFP FEN1Δ::HIS1 UPC2Δ::ARG4*	This study

Supplementary References

1. Fonzi, W.A., Irwin, M.Y. (1993). Isogenic strain construction and gene mapping in Candida albicans. *Genetics* **134,** 717-728.
2. Enloe, B., Diamond, A., Mitchell, A.P. (2000). A single-transformation gene function test in diploid *Candida albicans*. *J. Bac.* **182**, 5730-5736.
3. Hickman, M. A. et al. The 'obligate diploid' *Candida albicans* forms mating-competent haploids. *Nature* **494**, 55-59 (2013).
