# Supplementary material for: Candida albicans gains azole resistance by altering sphingolipid composition
Source: Nat Commun. 2018 Oct 29;9:4495. doi: 10.1038/s41467-018-06944-1 (PMC6206040; doi:10.1038/s41467-018-06944-1)
Supplement: Supplementary file 3 — Description of Additional Supplementary Files [file 41467_2018_6944_MOESM3_ESM.pdf]

Description of Additional Supplementary Files

File Name: Supplementary Data 1
Description: Oligonucleotide primers used in this study.

File Name: Supplementary Data 2
Description: Genome-wide profiling of genes whose inactivation require for fluconazole susceptibility.
