# Supplementary material for: Candida albicans gains azole resistance by altering sphingolipid composition
Source: Nat Commun. 2018 Oct 29;9:4495. doi: 10.1038/s41467-018-06944-1 (PMC6206040; doi:10.1038/s41467-018-06944-1)
Supplement: Supplementary file 4 — Supplementary Data 1 [file 41467_2018_6944_MOESM4_ESM.docx]

**Supplementary Data 1. Oligonucleotide primers used in this study.**

| Primer Name | Sequence (5′-3′) |
| --- | --- |
| ARG4F | TCTTGAACGGGCACATAAAGAAATCG |
| ARG4R | GTCAGGGTTTTTCTTTTGTGGCATCA |
| PBLR | GACGGATTCGCGCTATTTAGAAAGAGA |
| URA3F | GAGTAATTACAAAGTACTAATAGGAATTGA |
| URA3R | AACATTTGACCCCCCTGTCATGATTTCTAG |
| URA5R | GATGATTTTATATCTACTGTTTTTGGCTGTAT |
| HIS1Uf | ATGGATTTAGTCAATCATTTACCAGACCGT |
| HIS1URA3Ur | TTAGTACTTTGTAATTACTCGAAGACACAATTTTC |
| HIS1URA3f | GAAAATTGTGTCTTCGAGTAATTACAAAGTACTAA |
| HIS1URA3r | CCACTTTCAACCAAAAAGGACCACCTTTGATTGTA |
| HIS1URA3Df | TACAATCAAAGGTGGTCCTTTTTGGTTGAAAGTGG |
| HIS1Dr | TTAAACTCTACAATTTGATATCTCGAGTACC |
| ERG3Uf | CAATCATTTTCACAAATAGATTTCCCAAGT |
| ERG3URA3Ur | TTAGTACTTTGTAATTACTCCATAACTGGAATGGC |
| ERG3URA3f | GCCATTCCAGTTATGGAGTAATTACAAAGTACTAA |
| ERG3URA3r | CCAGTGTAACCATCTAAGGACCACCTTTGATTGTA |
| ERG3URA3Df | TACAATCAAAGGTGGTCCTTAGATGGTTACACTGG |
| ERG3Dr | CTCTATCGTCAACTTTACCTTCAACTTCTCC |
| PMC1Uf | CGACAACAACTAATGATGTTGAACCATTTC |
| PMC1URA3Ur | TTAGTACTTTGTAATTACTCCCAGTTAAAGCTGAT |
| PMC1URA3f | ATCAGCTTTAACTGGGAGTAATTACAAAGTACTAA |
| PMC1URA3r | TAATAACGGCGTTACAAGGACCACCTTTGATTGTA |
| PMC1URA3Df | TACAATCAAAGGTGGTCCTTGTAACGCCGTTATTA |
| PMC1Dr | CATATTCGGAATTTTCAAATGCAGTAGAAT |
| C5_04050WUf | GCATATAAGAGTGGATGGATATTTGTCACTC |
| C5_04050WURA3Ur | TTAGTACTTTGTAATTACTCATCACATGCTTGACA |
| C5_04050WURA3f | TGTCAAGCATGTGATGAGTAATTACAAAGTACTAA |
| C5_04050WURA3r | AGTGATGATCTGGTTAAGGACCACCTTTGATTGTA |
| C5_04050WURA3Df | TACAATCAAAGGTGGTCCTTAACCAGATCATCACT |
| C5_04050WDr | CTGTCCCATCTGGGTTGATTTCTTTC |
| C6_03800CUf | TTCCAGTTTGGCAATCGATAGTGATAGT |
| C6_03800CURA3Ur | TTAGTACTTTGTAATTACTCACATCAGCATATGAC |
| C6_03800CURA3f | GTCATATGCTGATGTGAGTAATTACAAAGTACTAA |
| C6_03800CURA3r | AATATTCATTGATTCAAGGACCACCTTTGATTGTA |
| C6_03800CURA3Df | TACAATCAAAGGTGGTCCTTGAATCAATGAATATT |
| C6_03800CDr | AGTAAGATTTACAAATCATGGCTGTTGCC |
| MVB12Uf | TAAGTGATTTACATATAGCGATACTGGCAAAG |
| MVB12URA3Ur | TTAGTACTTTGTAATTACTCGTCTTGGTGGCTGAA |
| MVB12URA3f | TTCAGCCACCAAGACGAGTAATTACAAAGTACTAA |
| MVB12URA3r | TTCACGAAGTGATCGAAGGACCACCTTTGATTGTA |
| MVB12URA3Df | TACAATCAAAGGTGGTCCTTCGATCACTTCGTGAA |
| MVB12Dr | TTGTTTTACCAGCCACAACAACAGATAAGT |
| NOT5Uf | ATAAGATTAATGCCACAGAATCAGCTTCAC |
| NOT5URA3Ur | TTAGTACTTTGTAATTACTCTTCTAACTGATCGTA |
| NOT5URA3f | TACGATCAGTTAGAAGAGTAATTACAAAGTACTAA |
| NOT5URA3r | TGCACTTGTTATTGGAAGGACCACCTTTGATTGTA |
| NOT5URA3Df | TACAATCAAAGGTGGTCCTTCCAATAACAAGTGCA |
| NOT5Dr | ATGCTATCTATGCCATCGCCATAATAAAA |
| FEN12f | CAATTTTTGATTTCGTTGCTTCAAAACT |
| FEN12URA3Ur | TTAGTACTTTGTAATTACTCAAGCATTAGGAGAAC |
| FEN12URA3f | GTTCTCCTAATGCTTGAGTAATTACAAAGTACTAA |
| FEN12URA3r | TGCAGTGGCTCCATGAAGGACCACCTTTGATTGTA |
| FEN12URA3Df | TACAATCAAAGGTGGTCCTTCATGGAGCCACTGCA |
| FEN12r | GGCTTTCTTGACTTTTTTAGATGCATTACC |
| FMP27Uf | TATCGGTAGGGTCATTGAGATTTAGACTATG |
| FMP27URA3Ur | TTAGTACTTTGTAATTACTCTTCTGCCTGTGATGG |
| FMP27URA3f | CCATCACAGGCAGAAGAGTAATTACAAAGTACTAA |
| FMP27URA3r | ATCCAGCAGCATCAGAAGGACCACCTTTGATTGTA |
| FMP27URA3Df | TACAATCAAAGGTGGTCCTTCTGATGCTGCTGGAT |
| FMP27Dr | TTAGGTTCACAATGTCGAAGGACTAATCG |
| C7_02530CUf | CTTCATCTTTAGGATCTAGTGGATCTAAGGG |
| C7_02530CURA3Ur | TTAGTACTTTGTAATTACTCTAACATTGAATTGGT |
| C7_02530CURA3f | ACCAATTCAATGTTAGAGTAATTACAAAGTACTAA |
| C7_02530CURA3r | TGGTAGTGGTAGTGGAAGGACCACCTTTGATTGTA |
| C7_02530CURA3Df | TACAATCAAAGGTGGTCCTTCCACTACCACTACCA |
| C7_02530CDr | TTAATATTCCAACTTTTCAAATCAGATCGATA |
| FGR32Uf | AACAATCTACTTCAATCTCAATCCCAGAAC |
| FGR32URA3Ur | TTAGTACTTTGTAATTACTCATGCAGAACTGTCAG |
| FGR32URA3f | CTGACAGTTCTGCATGAGTAATTACAAAGTACTAA |
| FGR32URA3r | CTGGGATGTTGTCATAAGGACCACCTTTGATTGTA |
| FGR32URA3Df | TACAATCAAAGGTGGTCCTTATGACAACATCCCAG |
| FGR32Dr | AACTGATTCAAAGGGCTTGAATCACGA |
| FEN12HIS1Ur | CACTACCAAATTACTACTAAAAGCATTAGGAGAAC |
| FEN12HIS1f | GTTCTCCTAATGCTTTTAGTAGTAATTTGGTAGTG |
| FEN12HIS1r | TGCAGTGGCTCCATGTGGGTACACAAGTTGAACTC |
| FEN12HIS1Df | GAGTTCAACTTGTGTACCCACATGGAGCCACTGCA |
| LAG1f | TTGGTTTCCTGCTTTCCCATTGACATA |
| LAG1SAT1Ur | ATTATTCTCTAGTTTTGACGCTACCTGATTAATTC |
| LAG1SAT1f | GAATTAATCAGGTAGCGTCAAAACTAGAGAATAAT |
| LAG1SAT1r | CCATCACTTCAAGTTGACCACCTTTGATTGTAAAT |
| LAG1SAT1Df | ATTTACAATCAAAGGTGGTCAACTTGAAGTGATGG |
| LAG1r | GTAATTGAGTTGAAGTTGACATAGTGGTGG |
| LAG1ARG4Ur | TACTCTTGAAAAATCTTACTCTACCTGATTAATTC |
| LAG1ARG4f | GAATTAATCAGGTAGAGTAAGATTTTTCAAGAGTA |
| LAG1ARG4r | CCATCACTTCAAGTTATTGGAGTACAAGGTATCTC |
| LAG1ARG4Df | GAGATACCTTGTACTCCAATAACTTGAAGTGATGG |
| UPC2f | GAGATCTTGATGTCATTAGTTTCAGAAGCA |
| UPC2SAT1Ur | ATTATTCTCTAGTTTTGACGCAATGAACTTGATTC |
| UPC2SAT1f | GAATCAAGTTCATTGCGTCAAAACTAGAGAATAAT |
| UPC2SAT1r | GGTATTGACCTGTCAGACCACCTTTGATTGTAAAT |
| UPC2SAT1Df | ATTTACAATCAAAGGTGGTCTGACAGGTCAATACC |
| UPC2r | GTTGTTGAAAGATCTTTCGTTATCGTGTGT |
| UPC2ARG4Ur | TACTCTTGAAAAATCTTACTCAATGAACTTGATTC |
| UPC2ARG4f | GAATCAAGTTCATTGAGTAAGATTTTTCAAGAGTA |
| UPC2ARG4r | GGTATTGACCTGTCAATTGGAGTACAAGGTATCTC |
| UPC2ARG4Df  URA3ApaI  URA3SacII  PB[URA3]EcoRI  PB[URA3]PstI | GAGATACCTTGTACTCCAATTGACAGGTCAATACC  CCGGGCCCTTGGCCAAGCCTAGATCCC  TCCCCGCGGAAGGACCACCTTTGATTGTAAATAGT  CGGAATTCTTTAACCCTAGAAAGATAGTCTGCGTAAAAT  AACTGCAGTTAACCCTAGAAAGATAATCATATTGTGACG |
| HIS1ApaI  HIS1SalI  PBaseXhoI | CGGGCCCTTAGTAGTAATTTGGTAGTGAAAAC  ACGCGTCGACGGTACCGCGGTGGGTACACAAGTTGAACTCCCTTAT  CCGCTCGAGATGGACCCTAAGAAGAAAAGAAAAGTTGATCCAAAGAAAAAGCGTAAGGTTGATCCTAAGAAAAAGAGAAAGGTTATGGGTTGTTCTTTAGACGATGAGCATATC |
| PBaseBamHI | CGCGGATCCTCAGAAACAACTTTGGCACATA |
| PBLf | CGACCGCGTGAGTCAAAATGAC |
| PBLr | TCCAAGCGGCGACTGAGATG |
| Seq1 | CGCGCTATTTAGAAAGAGAGAG |
| CDC28 sense | TGGATTAGCTCGAGCATTTG |
| CDC28 antisense | CCAACAGACCACATATCTACCC |
| URA3 sense | TGCTGATATTGGTAATACCGTGAAG |
| URA3 antisense | TGGCTCTTGGTTGGTGGTGGTTTCT |
| PBase sense | CCAGGGGCTCATTTGACCAT |
| PBase antisense | ACTCCGTTGGTCTGTGTTCC |
| ERG11 sense | ACCATTTGGTGGTGGTAGACA |
| ERG11 antisense | AGGGTCAGGCACTTTATAACCA |
| ERG3 sense | TACCGCTTGTCACACTGTCC |
| ERG3 antisense | CCCAAAGAGTAGTGAATTGACCG |
| UPC2 sense | TGCCACCACTGTTCCTCCTA |
| UPC2 antisense | TTATCGGCACTGTAGGCTGC |
| LAG1 sense | TGGTCAAGCAGCATTTTGGG |
| LAG1 antisense | ACAGCCAATCCCATCCAAGT |
| AUR1 sense | AGCTGCTCCTCCATGGTACA |
| AUR1 antisense | CAGGAGACCCGTGCATTGAA |
| MIT1 sense | ACTGATGAA CTGGCTCGTGA |
| MIT1 antisense | GCGTCTGCTCGTTGAATAGG |
| IPT1 sense | TGATACTCCAGGTTATGCCGC |
| IPT1 antisense | ATGGGGGAAGCATGAAATCCA |
| PBseq | CACGACGCTCTTCCGATCTGCATGCGTCAATTTTACGCAGACTATCTTTCTA |
| N7XX | CAAGCAGAAGACGGCATACGAGATXXXXXXXXGTCTCGTGGGCTCGG ^a^ |
| TruPBseq | AATGATACGGCGACCACCGAGATCTACACTCTTTCCCTACACGACGCTCTTCCGATCT |
| Illumina-R | CAAGCAGAAGACGGCATACGAGAT |

^a^ ‘XXXXXXXX’ denotes the 8-nucleotide multiplex indexes.
